# Monitoring of the Parasite Load in the Digestive Tract of *Rhodnius prolixus* by Combined qPCR Analysis and Imaging Techniques Provides New Insights into the Trypanosome Life Cycle

**DOI:** 10.1371/journal.pntd.0004186

**Published:** 2015-10-23

**Authors:** Felipe de Almeida Dias, Barbara Guerra, Larissa Rezende Vieira, Hugo Diego Perdomo, Ana Caroline Paiva Gandara, Raquel Juliana Vionette do Amaral, Renata Estebanez Vollú, Suzete Araujo Oliveira Gomes, Flavio Alves Lara, Marcos Henrique Ferreira Sorgine, Emiliano Medei, Pedro Lagerblad de Oliveira, Didier Salmon

**Affiliations:** 1 Instituto de Bioquímica Médica Leopoldo de Meis, Universidade Federal do Rio de Janeiro, Rio de Janeiro, Brazil; 2 Centro Nacional de Biologia Estrutural e Bioimagem—CENABIO, Universidade Federal do Rio de Janeiro, Rio de Janeiro, Brazil; 3 Instituto de Microbiologia Professor Paulo de Góes, Universidade Federal do Rio de Janeiro, Rio de Janeiro, Brazil; 4 Departamento de Biologia Geral, Universidade Federal Fluminense, Rio de Janeiro, Brazil; 5 Instituto Oswaldo Cruz, Fundação Oswaldo Cruz, Rio de Janeiro, Brazil; 6 Instituto Nacional de Ciência e Tecnologia em Entomologia Molecular (INCT-EM), Rio de Janeiro, Brazil; Liverpool School of Tropical Medicine, UNITED KINGDOM

## Abstract

**Background:**

Here we report the monitoring of the digestive tract colonization of *Rhodnius prolixus* by *Trypanosoma cruzi* using an accurate determination of the parasite load by qPCR coupled with fluorescence and bioluminescence imaging (BLI). These complementary methods revealed critical steps necessary for the parasite population to colonize the insect gut and establish vector infection.

**Methodology/Principal Findings:**

qPCR analysis of the parasite load in the insect gut showed several limitations due mainly to the presence of digestive-derived products that are thought to degrade DNA and inhibit further the PCR reaction. We developed a real-time PCR strategy targeting the *T*. *cruzi* repetitive satellite DNA sequence using as internal standard for normalization, an exogenous heterologous DNA spiked into insect samples extract, to precisely quantify the parasite load in each segment of the insect gut (anterior midgut, AM, posterior midgut, PM, and hindgut, H). Using combined fluorescence microscopy and BLI imaging as well as qPCR analysis, we showed that during their journey through the insect digestive tract, most of the parasites are lysed in the AM during the first 24 hours independently of the gut microbiota. During this short period, live parasites move through the PM to establish the onset of infection. At days 3–4 post-infection (p.i.), the parasite population begins to colonize the H to reach a climax at day 7 p.i., which is maintained during the next two weeks. Remarkably, the fluctuation of the parasite number in H remains relatively stable over the two weeks after refeeding, while the populations residing in the AM and PM increases slightly and probably constitutes the reservoirs of dividing epimastigotes.

**Conclusions/Significance:**

These data show that a tuned dynamic control of the population operates in the insect gut to maintain an equilibrium between non-dividing infective trypomastigote forms and dividing epimastigote forms of the parasite, which is crucial for vector competence.

## Author Summary

Although the key aspects of the *T*. *cruzi* life cycle were described more than one century ago, the development and interactions of *T*. *cruzi* with its vector are poorly characterized. By dissection of different compartments of the triatomine gut (prototype *Rhodnius prolixus*) (*i*.*e*., AM, PM and H) at regular time intervals, we evaluated trypanosome development within the insect using an accurate qPCR assay. qPCR analysis of trypanosomal colonization and clearance dynamics in real-time were confirmed *in vivo* using both fluorescence and bioluminescence imaging, which revealed massive parasite lysis during the first 24 hours post-feeding (p.f.). After one week, the parasite succeeded in establishing a resident population in each compartment of the gut, albeit at varying levels. From one week after the onset of infection in the AM and PM, some resident forms agglomerated into rosettes, clustering in close association with the vector tissue and constituting potential parasite reservoirs of the bug. For the first time, we have described a methodology to accurately quantify parasites in the insect gut that would be a useful tool for evaluating the impact of RNAi silencing of insect genes during the course of infection by *T*. *cruzi*.

## Introduction

Chagas disease is considered to be a neglected major parasitic tropical illness in Latin America that affects 7–8 million people distributed in 21 endemic countries and is responsible for 12,000 deaths each year (http://www.who.int/mediacentre/factsheets/fs340/en/). This disease is now an increasing worldwide public health problem due to the migration of infected subjects to non-endemic, more developed regions, mainly North America and Europe, where they could potentially transmit the disease by either hemotransfusion, organ donation, or pregnancy. Many of the chronically infected people develop severe and irreversible cardiovascular, gastrointestinal, and/or neurological problems that lead to a loss of productivity and high costs of medical care [1–3].

The etiological agent of Chagas disease is the protozoan *Trypanosoma cruzi*, which is transmitted in endemic areas principally via hematophagous triatomine insects [4]. Among the 140 triatomine species, *Rhodnius prolixus* is one of the most important vectors of Chagas disease in Central and South America and was chosen by researchers several decades ago as a prototype for the study of Chagas transmission, principally due to its use as a model for studies of insect physiology and biochemistry since the 1930s [5]. While some of the major features of the *T*. *cruzi* life cycle were described more than one century ago, the full development of the parasite within its vector remains poorly characterized. During its complex life cycle, the parasite encounters drastic environmental changes (*e*.*g*., temperature and pH) that are accompanied by major morphological and biochemical changes and cycling switches between multiplicative and non-proliferative stages. When the triatomine feeds on an infected vertebrate host, the bloodstream trypomastigotes are ingested and differentiate in the anterior midgut (AM) predominantly into epimastigote forms [4]. These latter forms replicate principally in the posterior midgut (PM) and can attach to the perimicrovillar membranes that cover the intestinal cells [6]. Some of these epimastigotes attach to the hindgut (H) wall and differentiate into infective metacyclic trypomastigotes that are released in the excreta [7,8]. During insect feeding on the blood of the mammalian host, contaminated feces and urine are released on the vertebrate host skin and subsequently enter the bloodstream by scratching the bite wound or via the mucosa.

The analysis of the development and distribution as well as the quantification of *T*. *cruzi* in different segments of the insect gut have been mostly based on optical microscopy approaches. To date, no standardized assay has been available to quantify parasites that are present at low levels in the gut. The classical technique for counting, using a Neubauer hemocytometer, has been employed to quantify *T*. *cruzi* in whole homogenates or gut contents, as well as in excreta samples [9–12]. Although useful for rapid quantification and to make comparisons between samples containing high parasite loads, this method is neither accurate nor statistical for samples containing a low parasite load, which is a common occurrence in the field [9]. Indeed, samples containing less than one parasite per microliter are not scored using the Neubauer counting chamber. In addition, the quantification of parasites in gut samples by microscopic counting is frequently hampered by the presence of a high concentration of erythrocytes in the AM, heme aggregates in the PM and H, and urate crystals in the H. Moreover, it may be complicated to discriminate immobile parasites from tissue debris and to count the exact number of parasites that form rosettes. Recently, the use of bioluminescent *T*. *cruzi* has revealed the feasibility of following the parasite distribution *in vivo* within the insect gut, thus providing the possibility of observing biologically relevant features of the parasite life cycle involved in vector-parasite interactions [13]. Similarly, the use of GFP-tagged *T*. *cruzi* as a microscopic tool for biological studies in infected hosts has been previously reported [14].

In contrast to the classical techniques, molecular-based assays such as quantitative real-time PCR (qPCR) allow a more sensitive quantitative analysis because these techniques are able to specifically amplify the parasite DNA sequence target [15,16]. However, the amplification of DNA samples extracted from the intact triatomine gut by PCR may be inhibited by the co-purification of several potential PCR inhibitors [17] such as heparin (added to blood to avoid clotting during artificial infection), polysaccharides (*e*.*g*., [18] polysaccharides produced by the microbiota), hemoglobin (released by hemolysis in the AM), hemozoin (heme aggregate, similar to hematin, which is produced in the PM [19]) and possibly uric acid (an end-product of purine metabolism that is highly abundant in the H).

The first aim of the present study was to describe an extraction protocol that allows the isolation of PCR inhibitor-free DNA from gut homogenates of triatomine bugs and to develop and evaluate the efficiency of a highly sensitive and accurate qPCR assay for the quantification of *T*. *cruzi* loads in these samples. Here, by combining this highly sensitive and accurate qPCR strategy with qualitative imaging analyses (fluorescence microscopy and BLI detection), we provide new insight into the dynamics of insect gut colonization that reveals several key features of the life cycle of *T*. *cruzi*.

## Methods

### Ethics statement

All of the animal care and experimental protocols were conducted in accordance with the guidelines of the institutional animal care and use committee (Comissão de Avaliação do Uso de Animais em Pesquisa da Universidade Federal do Rio de Janeiro, CAUAP-UFRJ) and the NIH Guide for the Care and Use of Laboratory Animals (ISBN 0-309-05377-3). The protocols were approved by CEUA-UFRJ (Comissão de Ética no Uso de Animais da Universidade Federal do Rio de Janeiro) under registry #115/13. Technicians at the animal facility of the Institute of Medical Biochemistry Leopoldo de Meis (UFRJ) performed all of the rabbit husbandry under strict guidelines to ensure careful and consistent handling of the animals.

### Insects

The insects used herein were adult mated females obtained from a *Rhodnius prolixus* colony, maintained at 28°C and 75% relative humidity, and fed on New Zealand white rabbit blood.

### Parasites

*T*. *cruzi* epimastigotes (Dm28c clone and CL Brener) were grown in liver infusion tryptose (LIT) culture medium containing 10% heat-inactivated fetal bovine serum at 28°C. Luciferase- and GFP-expressing parasites were grown in LIT in the presence of 200 and 300 μg/ml geneticin, respectively. Epimastigote forms were obtained during the log growth phase. Trypomastigote (Dm28c clone) forms from cell culture were obtained using metacyclic trypomastigote parasites to infect LLCMK2 host cells.

### Generation of *T*. *cruz*i lineages expressing GFP and luciferase

*pTEX-GFP*–A fragment of 718 bp corresponding to the GFP coding region from plasmid pEGFP-C3 (Clontech Laboratories Inc., Mountain View, CA, USA) was amplified by PCR using the following oligonucleotides (For: 5´-GGGGGATCCATGGTGAGCAAGGGCGAGGAGCTGTT-3’ and Rev: 5´-GGGGGATCCTCACTTGTACAGCTCGTCCATGCCAGAGTGATC-3´) and cloned into the *BamH*I sites of the pTEX episomal expression vector [20]. The PCR reaction was performed using a PCR thermal cycler (model 9700, Applied Biosystems) for 30 cycles under the following conditions (94°C, 1 min; 55°C, 30 sec and 72°C, 1 min). The resulting supercoiled plasmid (100 μg) was transfected into mid-log phase *T*. *cruzi* epimastigotes (Dm28c) by electroporation using a gene pulser II electroporation system (Bio-Rad) as previously described [21]. Transgenic parasites were selected and maintained in complete LIT medium containing geneticin (150 μg/ml).

*pBS*:*THT-Luc-T–*pBS:THT–Luc–T integrative vector encoding firefly luciferase (a generous gift of David Engman, Northwestern University, Chicago) was used to achieve the stable integration of the luciferase gene into the trypanosomal tubulin locus. Ten micrograms of *Sal*I-linearized plasmid was transfected into mid-log phase *T*. *cruzi* epimastigotes (Dm28c) using the Human T cell nucleofector kit in an Amaxa transfection device (set to program TX-011). Parasites were selected for resistance to hygromycin (50 μg/ml after 24 hours, increased to 200 μg/ml during drug selection), and luciferase activity was assessed by serial dilutions of the parasites *in vitro*.

*pTREX-luc* Dm28c strain (epimastigote and trypomastigote forms) (a generous gift from Cristina Henriques and Wanderley de Souza, UFRJ) were grown in the presence of 200 μg/ml geneticin. pTREX-luc is an integrative vector that targets the ribosomal locus. Trypomastigote forms from cell culture were obtained using metacyclic trypomastigote parasites to infect LLCMK2 host cells.

### Gut microbiota cultivation

*Rhodococcus rhodnii*, the only cultivable bacterium that colonizes the gut of insects from our *Rhodnius prolixus* colony, was isolated from the AM of starved insects and grown in Luria-Bertani (LB) broth at 28°C with shaking (120 rpm). Estimation of bacterial concentration was performed by OD measurement at 600 nm using the Bioscreen C turbidimetric analyzer (Labsystems Oy, Helsinki, Finland). Bacteria were grown to log phase and harvested by centrifugation for posterior use in insect feeding. To establish axenic first instar nymphs, eggs were collected and washed with sodium hypochlorite (1%) for 40 seconds, and three times with sterile PBS. The eggs were laid to hatch in sterile vials and maintained at 28°C.

### Infection

The experimental insects were allowed to feed through a latex artificial membrane feeding apparatus on heparinized (2.5 units/ml) and heat-inactivated rabbit blood (heated at 56°C during 45 min) containing 10^7^ x ml^-1^ or 10^5^ x ml^-1^ parasites (epimastigotes or trypomastigotes) expressing either GFP or luciferase. For infection, the adults were starved for 28 days and the first instar nymphs were used 5–7 days after hatching. Where indicated, adult and nymph insects were infected with *T*. *cruzi* plus, respectively, 10^8–^10^9^/ml *R*. *rhodnii*, or 10^5^/ml *R*. *rhodnii*. In a specific set of experiments, the adult insects were fed with blood containing 1 x 10^7^ heat-killed (65°C, 2 h) parasites per ml. Only fully engorged insects were used in the experiments. After infection, the insects were maintained as described above.

### Epifluorescence microscopy

The insects fed with blood containing 10^7^ GFP-expressing epimastigotes/ml were dissected in phosphate-buffered saline (PBS, pH 7.2), mounted between slides and coverslips and observed without fixation. Alternatively, the AM was opened with tweezers and mounted between slides and coverslips to allow a better analysis of the digestive cells in the apical region. Differential interference contrast (DIC) and fluorescence microscopy were performed using a Zeiss Axiobserver Z1 microscope with a 100 W mercury lamp filtered by a Zeiss-10 filter set for GFP (excitation–BP 450–490; beam splitter–FT 510; emission–BP 515–565). Images were acquired using a monochromatic Axiocam MRC5. Where indicated, color fluorescence image acquisition was performed using a Zeiss Axioplan 2 fluorescence microscope with a 100 W mercury lamp filtered by a Zeiss-09 filter set (excitation BP 450–490; beam splitter FT 510; emission LP 515) and acquired by a 10–24 mm Fujicolor Superia X-Tra film 400 ASA exposition. All of the obtained images were processed equivalently using Adobe PhotoShop.

### Bioluminescent imaging

Insects infected with parasites expressing luciferase, or their dissected guts, were monitored, and images were acquired throughout the experimental period using the bioluminescent imaging system (IVIS-Lumina; Caliper, Hopkinton, MA). Images were analyzed using the Living Image 3.2 software. The imaging time was 5 minutes. Adult insects were injected in the hemocoel with 3 μl of luciferin substrate (VivoGlo luciferin *in vivo* grade (Promega), 15 mg/ml) using a Hamilton syringe prior to imaging. First instar nymphs were injected with 138 nl of the same solution using a nano-injector (Nanoject, Drummond) with glass capillary needles. *In vivo* experiments: fifteen minutes after injection, the insects were imaged in the dorsal position. *Ex vivo* experiments: fifteen minutes after injection, the insects were dissected, and the guts were imaged.

### DNA extraction from digestive tract samples of infected insects

Several DNA extraction methods were tested to purify DNA from digestive tract samples of infected insects (phenol-choroform extraction, QIAamp DNA mini kit (Qiagen) and Cetyl trimethyl ammonium bromide (CTAB)). The classical phenol-chloroform method used to purify DNA from blood samples and animal tissues is not suitable for DNA purification from the insect gut because of its high content of hemozoin, which is invariably found in the PM and H samples harvested from days 3 to day 21 p.f.. As previously reported, even during blood sample processing, the presence of PCR inhibitors that are co-purified with DNA can affect the efficiency of the qPCR reaction and result in an underestimation of the parasite loads [22]. Another approach was attempted using a kit based on silica-DNA binding technology (QIAamp DNA mini kit, QIAGEN). The results showed that the efficiency of the DNA recovery was severely reduced by the presence of hemozoin, producing brownish DNA pellets, as confirmed by the false-negative PCR results. Only the latter technique, using CTAB, provided reproducible results, potentially due to the selective precipitation of DNA and elimination of hemin/hemozoin and polysaccharides. After infection with parasites constitutively expressing GFP, the AM, PM and H were extracted from cold*-*anesthetized insects at the indicated time points p.i.. Each gut section was homogenized separately in 1 ml of Solution A (1.5% CTAB; 2 M NaCl; 10 mM EDTA; 100 mM sodium acetate, pH 4.6). After homogenization, a 100-μl aliquot was transferred to a 2-ml microfuge tube containing 900 μl of Solution B (Solution A containing 10 μg salmon sperm DNA as DNA carrier, 10 ng of plasmid pLew82 (ble) [23] and 125 μg RNAse A per each 900-μl aliquot) and vortexed for 2 min. The samples were then heated for 30 min at 65°C for RNAse A activity. After the addition of 500 μl of chloroform, the samples were heated for 2 h at 65°C. Following this step, the samples were incubated for 10 min at RT and centrifuged at 9,000 × *g* at 25°C. After transferring a 600-μl aliquot of the aqueous phase to a new tube, 300 μl of chloroform was added to the samples followed by vortexing for 2 min. The samples were then incubated at RT for 10 min and centrifuged at 20,000 × *g* for 10 min at 25°C. A 400-μl aliquot of the aqueous phase of each sample was precipitated at -20°C for 1 hour after the addition of 1 μl of 20 mg/ml glycogen solution (Sigma-Aldrich) (or salmon sperm DNA (ssDNA), which provided comparable results), 40 μl of 3 M sodium acetate (pH 5.2) and 1 ml of ice-cold ethanol. The samples were then centrifuged at 20,000 × *g* at 4°C for 10 min. The DNA pellet was washed twice with cold 70% ethanol, dried at RT and resuspended in 50 μl of nuclease-free water.

### Parasite standard calibration curve

*T*. *cruzi* DNA standards were obtained from either parasite-free whole gut homogenates from insects on day 7 p.f. or rabbit blood that was serially spiked with 10^7^ parasites diluted 10-fold with nuclease-free water and containing 10 μg/ml salmon sperm DNA (ssDNA) to cover a range between either 10^5^ and 0.001 or 2.5 x 10^6^ and 2.5 parasite equivalents for the Dm28c and CL Brener lineages, respectively, per 5 μl of sample added to the reaction mixture.

### Plasmid standard calibration curve

The standard calibration curve for the internal loading control (pLew82) [23] containing the *ble* resistance gene was performed using the same reconstituted samples as used for the parasite calibration curves; insect gut homogenates or rabbit blood samples were spiked with 10 ng of plasmid and serially diluted 10-fold with nuclease-free water containing 10 μg/ml salmon sperm DNA (ssDNA) to cover a range between 10 and 0.01 ng of plasmid.

### Heterologous internal loading control

All of the samples were extracted in the presence of 10 ng of pLew82 plasmid used as a heterologous internal standard. The standard calibration curve for the internal loading control was used to determine the percentage of DNA recovery after extraction.

### Real-time PCR and parasite quantification

qPCR was performed using a StepOnePlus real-time PCR system (Applied Biosystems) with Power SYBR Green PCR master mix (Applied Biosystems) in a final volume of 15 μl. The PCR reactions consisted of 5 μl of DNA sample and 500 nM of *T*. *cruzi* repeat DNA-specific primers [15], which amplify a 182-bp amplicon from tandemly repeated satellite DNA. Specific *T*. *cruzi* DNA-oligonucleotides were TcFw (5'-GCTCTTGCCCACAMGGGTGC-3'), where M = A or C, and TcRv (5'-CCAAGCAGCGGATAGTTCAGG-3'). In parallel, reactions containing 5 μl of DNA sample and 500 nM of oligonucleotides designed to PCR amplify a 148-bp fragment of the *Sh ble* gene from plasmid pLew82 were performed using the following set of primers: BleFw (5'-CAAGTTGACCAGTGCCGTTC-3') and BleRv (5'-GCTGATGAACAGGGTCACG-3') as loading control. The qPCR conditions used for both primers pairs were as follows: 10 min at 95°C followed by 40 cycles of 15 s at 95°C and 15 s at 60°C (data collection). The amplification step was followed by a melting curve analysis to ensure that a single product was amplified. The data were analyzed with StepOne software v2.3. Negative controls for both primer pairs consisted of a reaction with no DNA. For each primer set, the efficiency of the amplification was determined using the following formula: Efficiency (E) = -1 + 10 ^(−1/slope)^. The protocols used for the qPCR experiments followed the Minimum Information Required for Publication of Quantitative Real-Time PCR Experiments (MIQE) guidelines [24].

### Normalization and quantification of parasite loads per gut section

Quantification of the parasite equivalents from DNA samples was calculated considering both the amplification curve of standard *T*. *cruzi* DNA and the percentage of DNA applied to the well. The results were normalized according to the heterologous internal standard amplification curve.

## Results

### Analytical sensibility of qPCR with CTAB-purified DNA for distinct phylogenetic *T*. *cruzi* lineages

Among the several extraction methods tested to purify the DNA from the insect gut (phenol-chloroform, QIAamp DNA mini kit (Qiagen) and CTAB), only the latter provided reproducible results and no inhibition of the PCR reaction. Therefore, this method was chosen to purify the DNA. Similarly, particular attention was paid to the concentration of heparin added to the blood as an anticoagulant for the extraction of plasma DNA due to its well-known inhibitory effect on the PCR reaction [17]. The use of citrate or EDTA as anticoagulants was discarded because of their toxicity to the insect, which becomes especially evident over long periods of time p.f..

**Fig 1:**
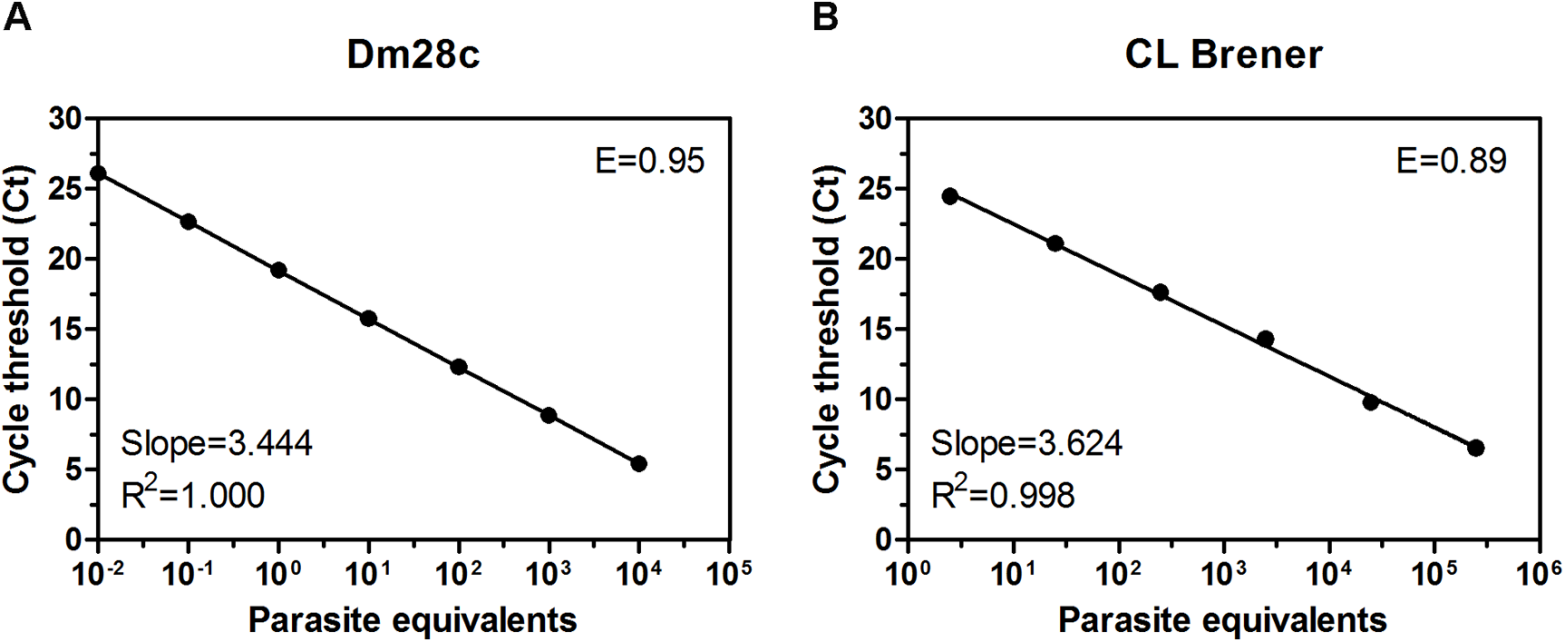
Representative standard qPCR calibration curves generated from 10-fold serial DNA dilutions of blood spiked with *T*. *cruzi* Dm28c (A) or CL Brener (B). The results are expressed as parasite equivalents/50 ng DNA. The slope and regression coefficient of the curves as well as the amplification efficiency for each parasite lineage are indicated.

As expected, the DNA recovery from the different gut samples was highly variable even in the presence of ssDNA as the DNA carrier, requiring a heterologous internal loading control to normalize the DNA recovery for each sample extraction. We first assessed the efficiency of the PCR amplification of the internal standard using serial dilutions of plasmid containing the *ble* resistance gene spiked into heparinized rabbit blood for comparison to that of the purified *T*. *cruzi* DNA targeting repetitive satellite sequences [15]. Highly similar amplification efficiency percentages were obtained for both amplicons (E = 0.95 for satellite DNA vs. E = 0.96 for *ble*), thus validating the use of the internal standard for qPCR (S1 Fig). Therefore, the sensitivity of the *T*. *cruzi* satellite DNA-based qPCR was determined by serial dilution of purified DNA from Dm28c (TcI) and CL Brener (TcVI) reference stocks after normalization by plasmid DNA spiked into the blood lysate (Fig 1). The qPCR provided a detection limit of 1 and 0.01 parasite equivalents (for a cycle threshold (Ct) number of approximately 25) for the CL Brener and Dm28c lineages, respectively. The parasite concentrations lower than 0.01 were still detectable but at a higher Ct value (approximately 30), which was close to the detection limit background. Dynamic ranges, amplification efficiencies and *R* ^2^ values (coefficient of determination) were, respectively, 0.01−10^4^ and 1−10^5^ parasite eq., 89 and 95% and 0.998 and 1.000, for the CL Brener and Dm28c lineages. To determine whether some trace of PCR inhibitor isolated from the total gut homogenates was able to affect both the PCR efficiency and sensitivity, we performed qPCR amplifications for Dm28c by replacing blood DNA with tissue-specific insect homogenates of the entire digestive tract (AM, PM and H) dissected one week after the blood-meal spiked with various parasite quantities (S2 Fig). The dynamic range, amplification efficiency and *R* ^2^ value were, respectively, 0.001−10^4^ parasite eq., 92% and 1, which were similar to those provided by the blood DNA extract. Thus, standard curves with a similar high sensibility were generated independently of the DNA origin, validating the robustness of the qPCR assay.

These data revealed that the use of the Moser-modified primers [15,25] designed to amplify satellite DNA from different *T*. *cruzi* lineages purified from blood or insect gut homogenates, combined with an internal standard (*ble*), successfully produced an accurate standard parasite calibration curve in both reference stocks. Thus, this methodology provides an optimized reliable qPCR assay that is adapted to quantify parasite DNA in the insect gut.

### Quantitative monitoring of *T*. *cruzi* load in the *R*. *prolixus* digestive tract

**Fig 2:**
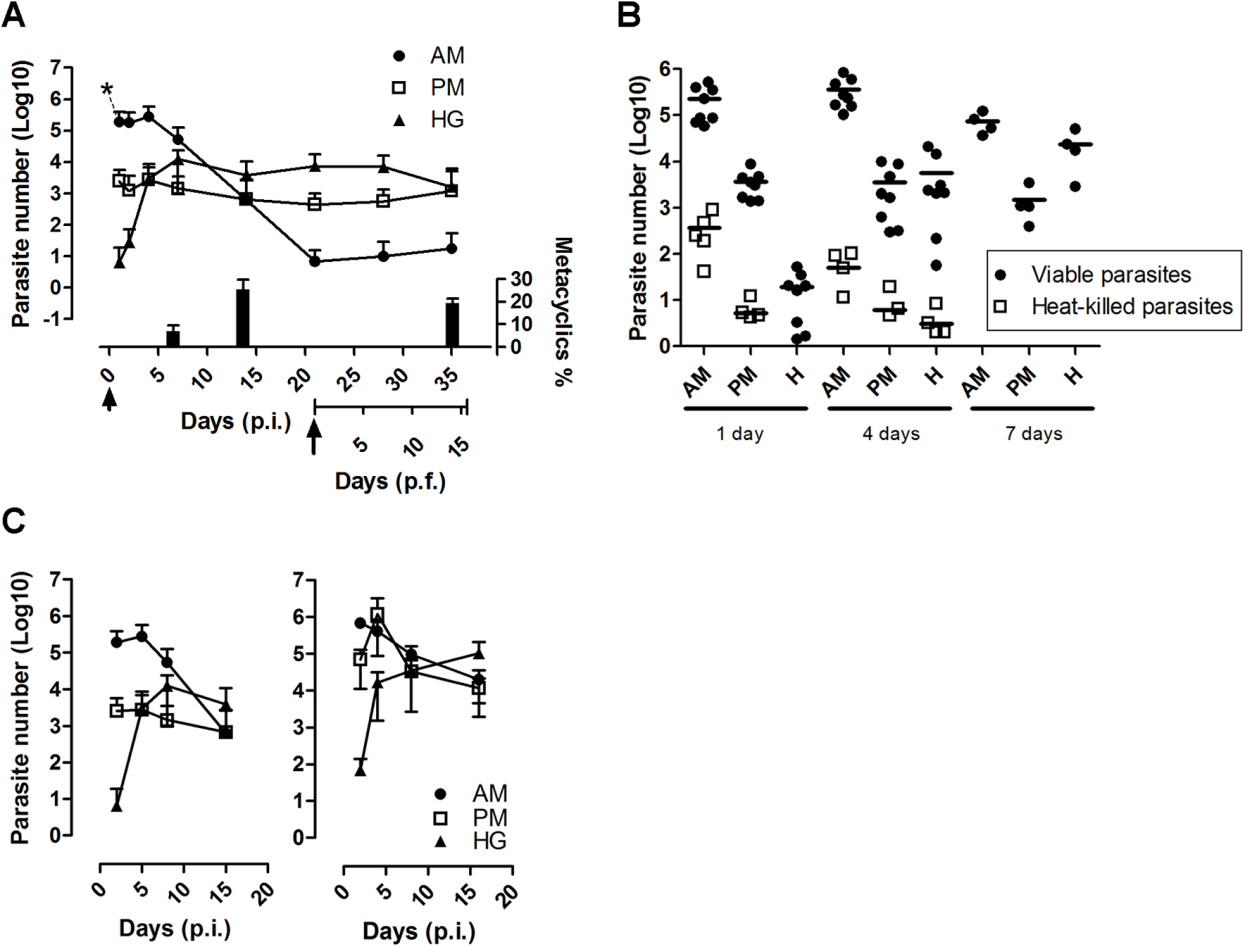
Real-time monitoring of *T*. *cruzi* loads in the different gut segments after *R*. *prolixus* infection. (A) Follow-up of parasite development in the anterior midgut (AM), posterior midgut (PM) and hindgut (H) post-infection (p.i.) and post-feeding (p.f.). Adult insects were fed with 10^7^ cells/ml of blood. The arrowhead and arrow indicates, respectively, the time of the initial infection and refeeding with a blood meal without parasites (21 days p.i.). Each time point represents three independent experiments (n≥8). The percentage of metacyclic trypomastigote forms (mean ± SE; n = 10) determined in the hindgut contents at 7 and 14 days p.i. and 14 days p.f. are indicated. (B) Monitoring of the DNA clearance of heat-killed parasites. Infections were performed with live parasites or parasites killed by incubation at 65°C during 2 hours before injection. n≥4 for each time point. (C) Comparison between the gut colonization of *R*. *prolixus* by *T*. *cruzi* Dm28c (left panel) and CL Brener (right panel). Each time point represents three independent experiments (n≥8). At several time points, the insects were dissected, and total DNA was extracted individually from the different gut segments and used to assess the parasite number by qPCR.

We assessed the parasite load of bugs that were artificially fed on decomplemented infected blood containing 10^7^ epimastigotes (Dm28c)/ml and dissected after different periods of time over three weeks (Fig 2A). During the first 24 hours p.i., ~ 92% of the parasites were lysed, and most of the live trypanosomes (1.9 x 10^5^ cells corresponding to 98.69% of the live parasites) were present in the AM while a resident population started to colonize the PM (1.29%). At day 4 p.i., the parasite population started to colonize the H, reaching a higher density at day 7 p.i. (~ 10^4^ parasite equivalents), while approximately 6.8% ± 2.78 (SE) (n = 5) of the total cells counted were metacyclic parasites (Fig 2B). At day 14 p.i., the proportion of metacyclic parasites increased and represented 25.2% ± 4.22 (SE) (n = 10) of the total population. It is noteworthy that two weeks after refeeding, the proportion of metacyclic parasites remained stable at 19.1% ± 2.05 (SE) (n = 10) (Fig 2B). Concomitantly, to the increase in the number of parasites from the rectum, the population from the AM dropped to its lower level after three weeks p.i. (a few dozen parasites). In contrast, the trypanosome populations in both the PM and H remained constant over two weeks with 10^3^ to 10^4^ parasite equivalents, respectively (Fig 2A). In an attempt to characterize the clearance dynamics, infected bugs were fed on decomplemented blood at three weeks after infection, and the parasite number was assessed by qPCR over two weeks in each segment of the gut (Fig 2A, arrow). Surprisingly, the three populations maintained a similar level of parasites despite the diuresis and urine flux mobilized during this process. This relative constant level of parasite populations possibly reflects the tight interactions between the vector and parasite [6] and results of the equilibrium between the number of parasites generated by cell replication and the number of parasites released in the excreta. In both the AM and PM, a slight increase in parasite numbers was observed, suggesting the presence of parasite nests that served as reservoirs (see results below).

The massive lysis event observed 24 hours after infection raised the possibility that the DNA provided by the dead parasites could contribute to an overestimation of the parasite load in the gut. To assess the amount of DNA template released from dead parasites remaining in the digestive tract that could eventually be amplified by qPCR, we performed a controlled experiment using insects fed with blood containing 10^7^ heat-killed parasites/ml (Fig 2C). In the AM and PM, 24 hours p.f. was sufficient to decrease the number of parasites to three logs below that obtained with live parasites, whereas at four days p.f., the contribution of the remnant DNA fell to only 4 logs below that of insects fed with live parasites. Thus, the initial contribution of DNA from lysed parasites represents only ~ 0.1% of the total number of parasites. Finally, the kinetics of parasite DNA degradation by insect gut nucleases showed that 7 days were necessary to completely remove all of the remnant DNA. These data suggest that the quantitative monitoring of the parasite load by qPCR is a reliable measurement for live parasites despite the large number of parasites that were lysed at the beginning of infection.

Next, we compared the colonization of the bug by the Dm28c strain (left panel), which belongs to TcI, a group related to the sylvatic cycle, with that of CL Brener (right panel), another *T*. *cruzi* lineage, which belongs to TcVI, a group related to human/domestic cycle (Fig 2D). Similar profiles of parasite CL Brener loads were generated in the different compartments of the gut, although a significant increase in resistance to lysis was observed, especially in the PM where the relative number of parasites reached 10^6^ on day 4 p.i., *i*.*e*., a difference of two and half logs compared to that observed for the Dm28c lineage (5 x 10^3^ parasites). After two weeks, the parasite load became stable in all of the gut compartments and reached a maximal value in the H (approximately 10^5^ cells). Altogether, these results show that the qPCR methodology can be used in a straightforward manner to study the colonization dynamics in insects infected by different parasite lineages.

### Monitoring of trypanosome infection using fluorescence and bioluminescence imaging

**Fig 3:**
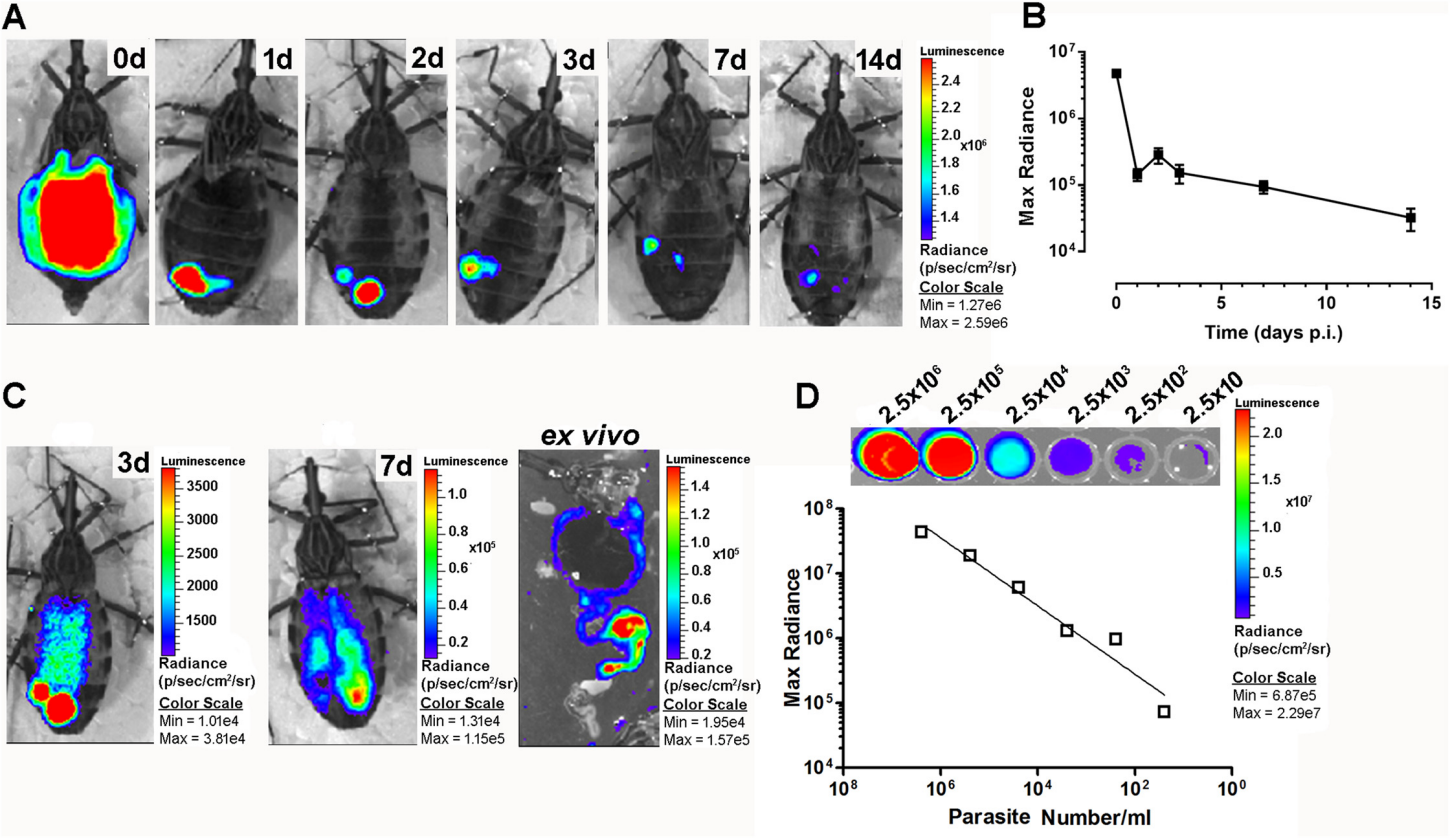
Real-time *in vivo* BLI of *Trypanosoma cruzi* infection in *R*. *prolixus*. (A) Insects were infected with *T*. *cruzi* Dm28c epimastigotes constitutively expressing luciferase, and time-course monitoring of infection was assessed for two weeks by BLI. (B) Quantification of the luminescence signal emitted by the infected insects at the times indicated in A. (C) *In vivo* luminescence evaluation of insects at day 3 and 7 p.i. and *ex vivo* at day 7. (D) Correlation between the bioluminescence emission measured in each well and parasite number. The images are representative of at least 24 insects analyzed at each time point in three independent experiments. The images of all of the insects analyzed at each time point were used to quantify the mean of the emitted luminescence signal.

**Fig 4:**
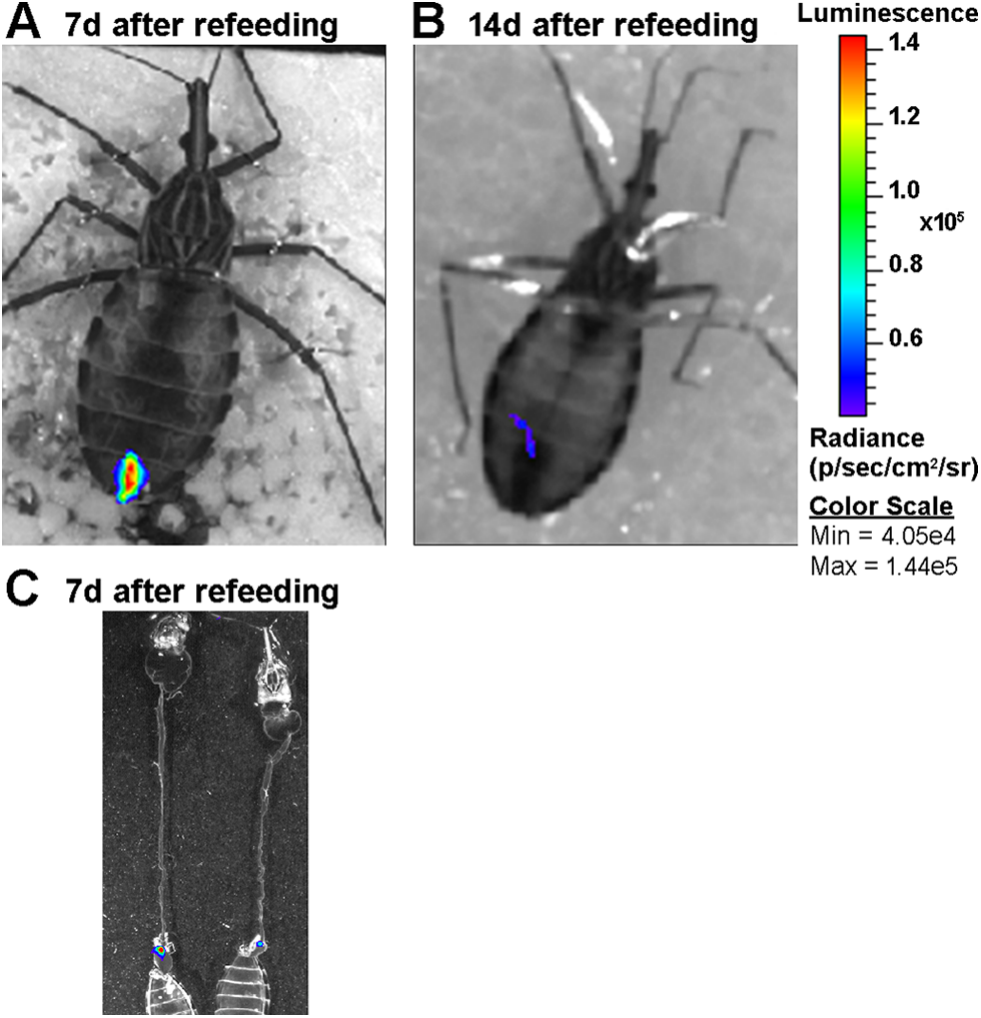
BLI of infected insects after feeding. *In vivo* (A, B) and e*x vivo* (C) BLI showing that at one to two weeks after refeeding, most of the parasites remains densely packed in the rectum, as evidenced by the punctuated luminescent signal located at the end of the digestive tract.

**Fig 5:**
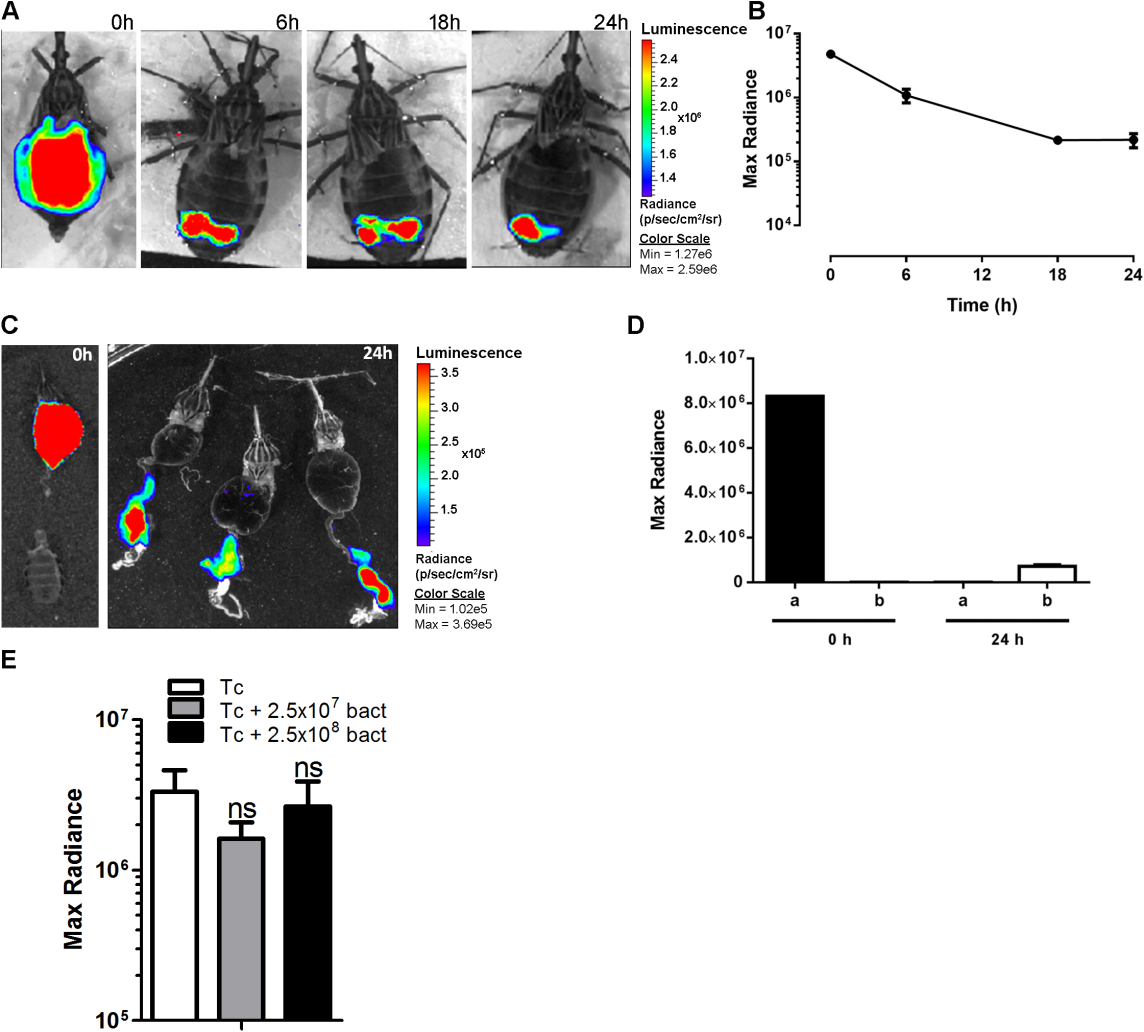
BLI time-course development of *T*. *cruzi* expressing luciferase in the insect digestive tract during the first 24 h p.i. (A) The images show the drastic reduction of the luminescence signal in the AM. (B) Quantification of the BLI signal emitted by the whole intestine obtained at different times as indicated in A. (C) *Ex vivo* BLI confirming that the signal was located exclusively in PM 24 hours p.i.. (D) Quantification of the BLI signal emitted by AM (a) and PM (b) at 0 and 24 hours after infection. (E) Effect of the microbiota on parasite lysis and BLI reduction. The insects were infected with parasites alone (Tc) or with parasites plus 2.5 x 10^7^ or 2.5 x 10^8^ *Rhodococcus rhodnii* per ml of blood. Two independent experiments (n = 16) were conducted, and the results were analyzed by one-way ANOVA.

**Fig 6:**
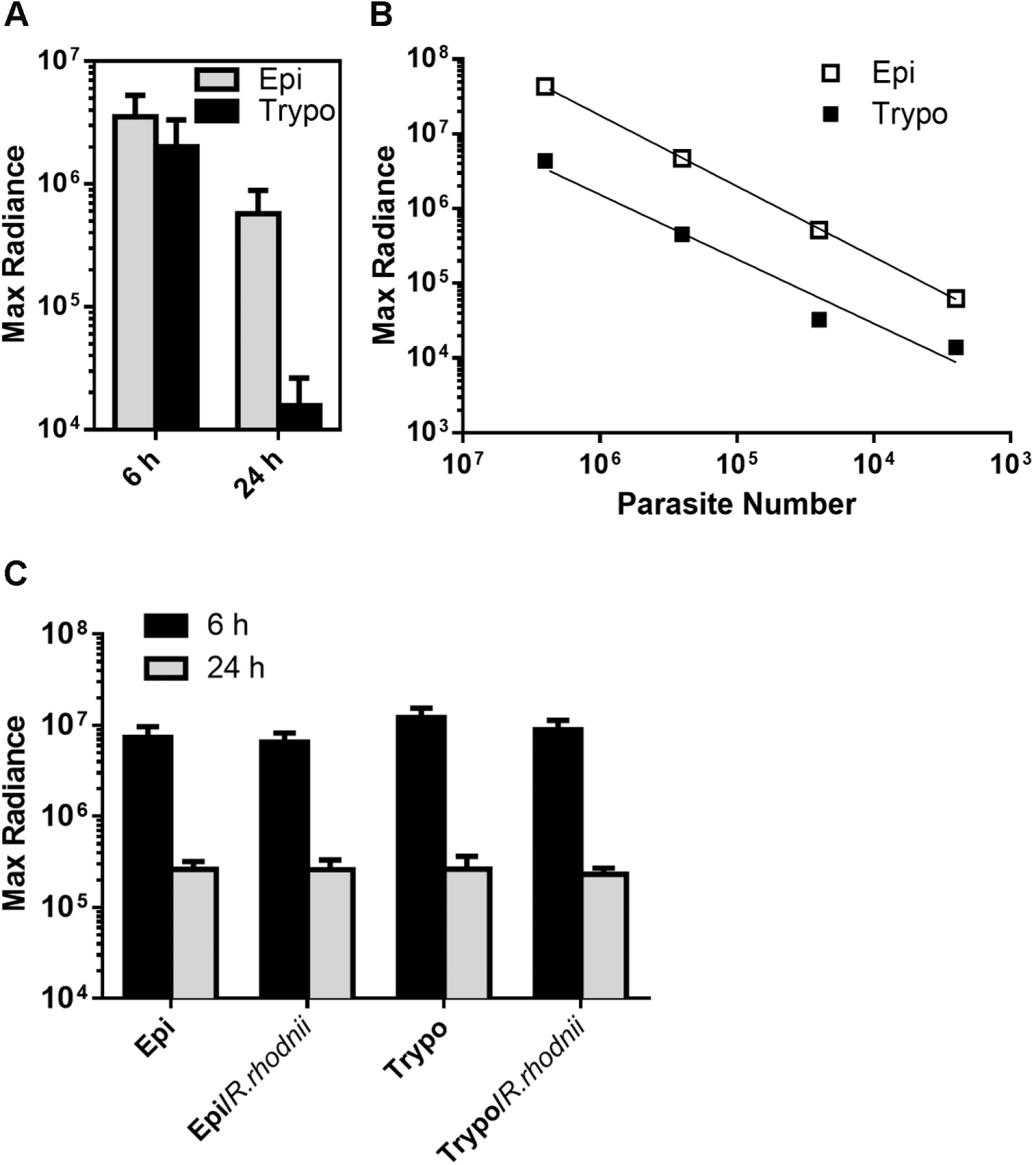
Comparison of time-course development of epimastigotes and trypomastigotes expressing luciferase in the digestive tract of *R*. *prolixus* during the first 24 h p.i., in the presence or absence of *R*. *rhodnii*. (A) Representative values of the luminescence emission for epimastigotes and trypomastigotes at 6 h and 24 h. Epi 6 h vs. Epi 24 h, P<0.01; Trypo 6 h vs. Trypo 24 h, P<0.0001. ANOVA followed by Tukey's multiple comparisons test. (B) Quantification of the BLI signal emitted by adult insects infected with various amounts of epimastigotes (Epi) or trypomastigotes (Trypo). (C) Quantification of the BLI signal emitted by gut microbiota-free first instar nymphs fed with epimastigote (Epi) or trypomastigote (Trypo) forms (10^7^ cells/ml), in the presence or absence of *R*. *rhodnii*. In each stage ± bacteria, 6 h vs. 24 h, P<0.001. ANOVA followed by Tukey's multiple comparisons test.

We qualitatively investigated the presence of live parasites in the digestive tract by BLI and fluorescence microscopy to compare these data with those obtained by qPCR. Using a Dm28c lineage constitutively expressing luciferase (pBS:THT-Luc-T integrative construct), AMs filled with parasites expressing saturating amounts of photons were observed immediately after the blood meal (red signal corresponding to radiance ~ 1.9 x 10^6^ p/sec/cm^2^/sr) (Fig 3A and 3B and S3 Fig) with some variability in photon emission reflecting the variable amount of blood ingested by the bugs (average of 242.9 ± 27.9 μl, n = 10 ± SD). After 6 hours, the light emission was significantly reduced in the AM, and most of the high intensity signal moved towards the posterior part of the insect abdomen. After 24 hours, the signal disappeared completely from the AM correlating with a large decrease in maximal radiance photon emission, and some red spots were distributed across the posterior end of the insect abdomen (Fig 3A and 3B). From days 3 to 7 p.i., we observed a decrease in the luminescent signal and, in some cases, a recolonization of the AM that could be explained by either dividing resident epimastigotes and/or parasites that had moved back from the PM (Fig 3C). Although the decrease in luminescence was proportional to the decrease in parasite number, as attested by the maximal radiance photon emission that correlated positively with the number of cells *in vitro* (Fig 3D) as previously reported [13], the use of BLI for the *in vivo* quantification of parasites in intact insects is clearly limited by the high detection threshold limit inherent to the technique. After 2 weeks, the signal was reduced to some punctuated parts of the PM. One to two weeks after refeeding, the luminescent signal disappeared completely from the insect midguts (Fig 4A and 4B) and was limited to the rectum (Fig 4C). Altogether, the BLI data corroborated the qPCR results showing a massive lysis that occurred during the first 24 hours p.i. and demonstrated that this process started very early, approximately 6 hours after the blood meal (Fig 5A and 5B). *Ex vivo* dissection confirmed the migration of the parasites to the PM and the complete absence of signal in the AM (Fig 5C and 5D). To analyze whether the cell lysis event was due to the bacterial endosymbionts of the triatomine bug, such as the actinomycete *Rhodococcus rhodnii*, which is the only cultivable gut bacterium found in our insect colony, we infected insects with blood containing either *T*. *cruzi* expressing luciferase or *T*. *cruzi* expressing luciferase plus different *R*. *rhodnii* concentrations (2 to 10 times higher than that in the starved AM of *R*. *prolixus* [26]). Although the bacterial load was considerably increased, no significant difference in the luminescent signal intensity was detected 24 hours p.i. between the two insect groups (Fig 5E). We also checked that heparin (2.5 U/ml), added to the blood as anticoagulant, had not effect on the *in vitro* growth of *R*. *rhodnii* (S4 Fig). These data suggested that under our experimental conditions, the gut microbiota did not appear to be involved in the drastic reduction of the epimastigote parasite number. We investigated whether such a decrease could be observed using trypomastigote parasites, which are supposed to be a more robust and resistant form that is adapted to the bloodstream of the vertebrate host [27] and corresponds to the stage that infects the insect vector under natural conditions. Using a Dm28c lineage constitutively expressing luciferase (pTREX-Luc integrative construct) in both epimastigotes and trypomastigotes in culture, we observed a large decrease in the luminescent signal after 24 h in both parasite life stages (Fig 6A). Whereas the trypomastigote parasites expressed approximately 10 times less luminescence than the epimastigote forms (Fig 6B), which is compatible with the 10-fold decrease of the transcription rate for RNA pol I in infective forms [28], from 6 h to 24 h, they displayed a luminescent signal reduction higher than that observed in epimastigotes, representing 0.77% and 16.2% of the ingested parasites, respectively. The observation that both the insect and bloodstream trypomastigote forms displayed a similar large decrease in photon emission indicated that the cold-shock was not responsible for the lysis of epimastigote parasites. We next investigated whether trypomastigote decrease was also unaffected by the gut microbiota, using first instar nymphs presenting the advantage to be naturally gut microbiota-free. Germ-free first instar nymphs were fed on decomplemented blood containing either *T*. *cruzi* trypomastigotes or epimastigotes (~ 10^5^ cells/nymph) expressing luciferase in the presence or absence of *R*. *rhodnii* (10^3^ bacteria/nymph) (Fig 6C and S5 Fig). Similar amplitude of decrease in the luminescent signal after 24 h was observed in both parasite life stages of nymphs infected or not by *R*. *rhodnii* arguing that gut microbiota is not involved in the parasite lysis of both stages during the first 24 h after blood meal.

**Fig 7:**
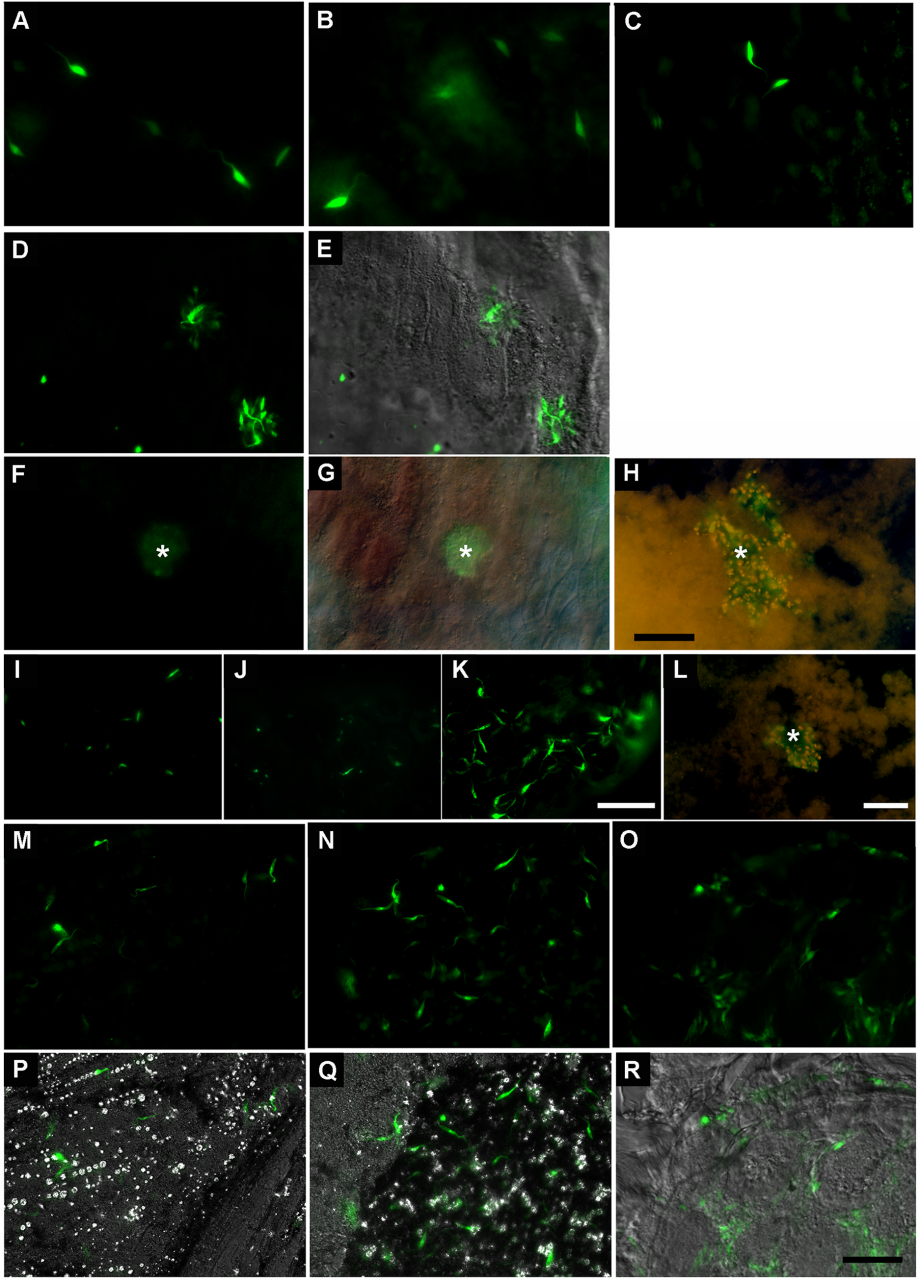
Fluorescence microscopy of digestive tracts extracted from insects infected with GFP-tagged parasites. The images show the presence of green fluorescent parasites colonizing the AM (A-H), PM (I-L) and H (M-R). (A-C) Fluorescence images of the AM showing free-swimming parasites at days 1, 2 and 7 p.i., respectively. (D-H) Fluorescence and DIC images showing parasite aggregates, at day 1 p.i. (D, E), and day 4 p.i. (F-H). The asterisks indicate aggregate of parasites associated to gut wall. (I-K) Fluorescence images of the PM at days 1, 2 and 7 p.i.. (L) Fluorescence image at day 4 p.i.. The asterisk indicates a cluster of parasites in the lumen of the PM. Fluorescence and DIC images of the H at days 4 (M, P), 7 (N, Q) and 21 (O, R) p.i., respectively. Scale bars: (A-H) 50 μm; (I-K) 50 μm; (L) 10 μm; (M-R) 50 μm. A-E, I-K, M-Q and F-H and L images were performed, respectively, using a Zeiss Axioplan 2 fluorescence microscope and a Zeiss Axioplan 2 fluorescence microscope.

Because of the high detection limit inherent to the BLI method, we used GFP-tagged parasites to check the relative parasite abundance and distribution, its morphology and the host tissue associations by fluorescence microscopy. At 24 hours p.i., the AM displayed a relative parasite density that was smaller than expected and was mainly represented by parasites near the epithelium. This phenomenon was due to both the presence of a large amount of intact erythrocytes that scattered the parasites and the globular form of the AM, which prevented the visualization of GFP-tagged parasites located in deeper sites (Fig 7A). S1 Video illustrates this feature showing parasites with a high fluorescence emission signal at the border of the epithelium and others deeper in the lumen of the midgut with a low emission signal [4,29]. From days 4 to 7 p.i., when the total parasite population decreased in the AM (Fig 7B and 7C), we observed free-swimming parasites and aggregates of trypanosomes forming rosettes (Fig 7D and 7E), some of these aggregates were associated with the gut wall (Fig 7F and 7G and 7H). In the PM, while the total parasite number remained unaltered during this period (Figs 2A and 7I and 7J), we detected a massive presence of parasites concentrated in some regions of the PM, frequently as trypanosome aggregates (Fig 7K and 7L). S2 Video shows that among the parasites scored in the PM, some were free-swimming while others appeared to be attached to the epithelium, possibly via the perimicrovillar membrane [6]. In the H, we observed a relative increase in the number of parasites from days 4 to 21 p.i. (Fig 7M and 7N and 7O), among which an important proportion was attached by the flagellum to the wall of the rectum (S3 Video) as previously reported by Schaub et al [30].

### Evaluation of dynamics of gut colonization by *T*. *cruzi* under natural conditions

**Fig 8:**
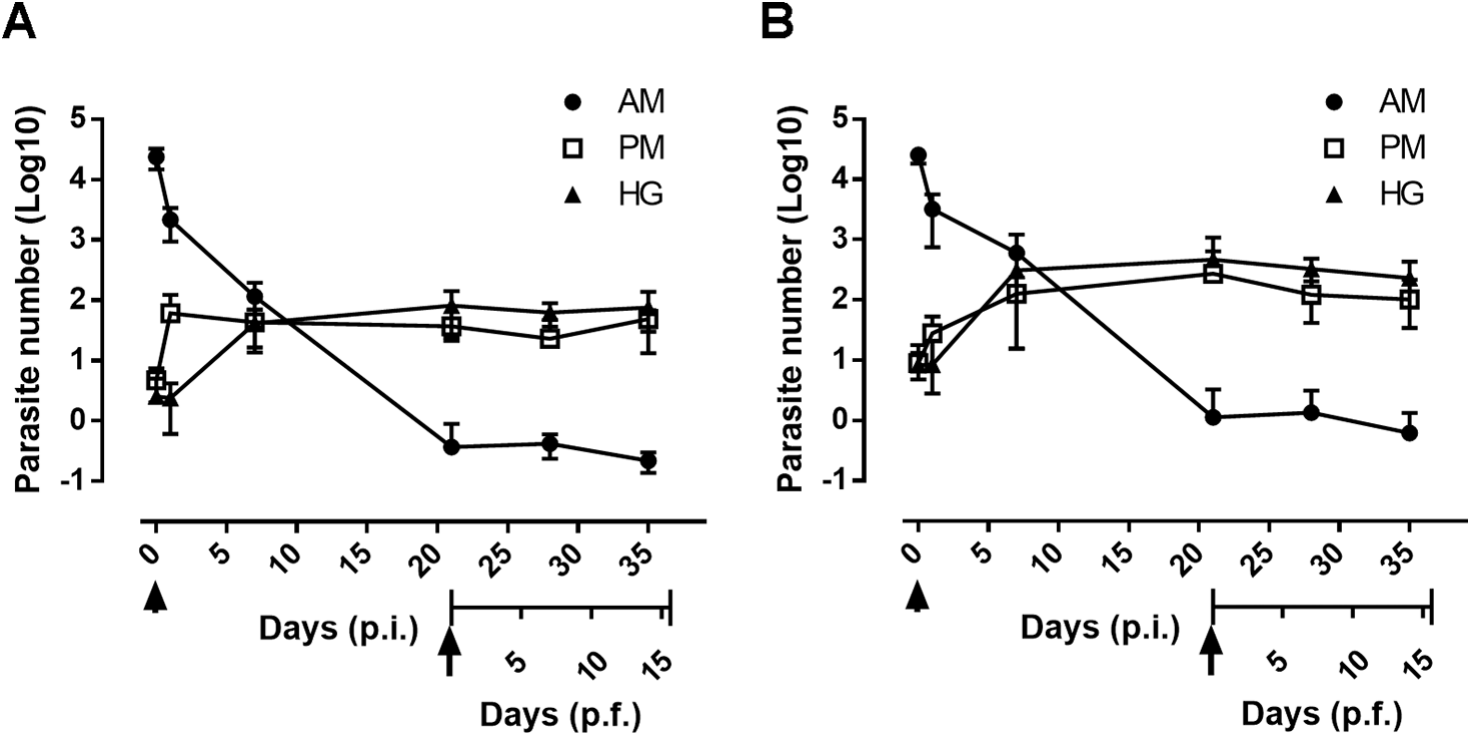
Real-time monitoring of *R*. *prolixus* gut colonization by *T*. *cruzi* in natural conditions. Follow-up of parasite development in the anterior midgut (AM), posterior midgut (PM) and hindgut (H) post-infection (p.i.) and post-feeding (p.f.) in epimastigotes (A) and metacyclics trypomastigotes (B). Adult insects were fed with 10^7^ cells/ml. The arrowhead and arrow indicates, respectively, the time of the initial infection a trypomastigotes and refeeding (21 days p.i.). At several time points, the insects were dissected, and total DNA was extracted individually from the different gut segments and used to assess the parasite number by qPCR. Each time point represents an experiment (n = 8).

The high optimization of qPCR leading to a high sensitivity of detection (detection limit of 1 and 0.01 parasite equivalents, respectively, for the CL Brener and Dm28c lineages), constitutes a proof of principle to assess the dynamics of natural infections of insects, which are frequently infected by low densities of bloodstream trypomastigotes (≤10^4^ parasites/insect). Therefore, we assessed the parasite load of insect adults artificially fed on decomplemented infected blood containing either 10^5^ trypomastigotes/ml or 10^5^ epimastigotes/ml (Dm28c) that were dissected after different periods of time over three weeks (Fig 8A and 8B). Similar profiles of parasite loads were observed throughout the different gut compartments of insects fed with epimastigotes or trypomastigotes. The insects were initially infected by approximately 2.5 x 10^4^ parasites and similarly to that previously observed, during the first 24 hours p.i., around 90% of these parasites were lysed (87.32% and 87.35% of trypomastigotes and epimastigotes, respectively) with a preferential colonization in the AM (corresponding to 97.18% and 98.9% of live epimastigotes and trypomastigotes, respectively). The resident population of epimastigotes that was initially present in the PM multiplied 12.9-fold in 24 h to colonize the PM while in the case the of insect fed by trypomastigotes we observed a lower increase of 3.1-fold, which is due to the differentiation of quiescent trypomastigotes to dividing epimastigotes. In the H, the parasite population of trypomastigotes reached its climax at day 7 p.i. (~ 10^2^ parasite equivalents) and stabilized over two weeks as it did in the PM with around 10^1^ to 10^2^ parasite equivalents of epimastigotes and trypomastigotes, respectively. After three weeks p.i., the AM populations had almost disappeared (approximately 1 parasite equivalent). Similarly to that observed previously, two weeks after refeeding insects infected by epimastigotes or trypomastigotes, a constant level of parasites was maintained within a similar range in each gut compartment, although a little bit higher in insects infected by trypomastigotes (Fig 8A and 8B arrow). Thus, the rate of insect infection by trypomastigotes is almost identical to that of epimastigotes displaying similar kinetics of colonization of the gut by low level of trypomastigotes leading to fully competent vector two weeks after refeeding. We can also conclude that the amplitude of the peaks of population fluctuating in the gut is function of the number of parasites that was initially ingested by the insect at the beginning of the infection.

## Discussion

In the present study, we report a highly sensitive and accurate qPCR strategy combined with qualitative and semi-quantitative imaging analyses for the quantification of the parasite load in each segment of the digestive tract and provide insight into the dynamics of insect gut colonization. While it is clear that blood digestion constitutes the principal function of the insect gut, very little is known about the proteins that are involved in the vector response after a blood meal and how they affect both trypanosome infection and gut colonization. During its journey through the insect gut, the parasite must adapt to several harsh environments that it will encounter, first in the AM where blood is stored and erythrocytes are lysed, followed by the PM were blood proteins are digested and heme is detoxified to generate large amounts of crystalline hemoglobin, and finally in the rectum where urate crystals accumulate and blood remnants are excreted. All of these by-products derived from blood digestion are susceptible to co-precipitate during DNA purification and inhibit further PCR reactions. In this respect, the use of CTAB for DNA extraction possesses several advantages over other purification methods (*e*.*g*., phenol-chloroform, silica-DNA binding technology): (i) it removes polysaccharides, such as chitin, a constituent of the hindgut procuticle, and bacterial polysaccharides supposed to be highly abundant in the digestive tract of *Rhodnius* (*e*.*g*., capsular polysaccharides produced by *Rhodococcus sp*.) [31–33]; (ii) the CTAB acidic buffer (pH 4.6) leads to the formation of hemin and hemozoin precipitates and thus do not interfere with both DNA recovery [34] and efficiency of PCR reaction.

Regarding the qPCR strategy developed in the present study, the primers designed by Moser and colleagues [25] and subsequently modified by Cummings and Tarleton [15] were found to be efficient for the accurate quantification of parasite loads in tissue independently of both the parasite lineages used (such as Dm28c (TcI), and CL Brener (TcVI)) and the parasite stage. In addition, the use of exogenous DNA plasmid rather than an internal marker (insect DNA marker) spiked into the insect gut samples was crucial to ensure the appropriate normalization for accurate quantification of parasite loads due to the occurrence of individual differences in the number of gut epithelial cells.

Our results showed that the qPCR methodology developed herein allowed the determination of parasite numbers in ranges far below that obtained using the Neubauer chamber for cell counting (threshold limit ~ 10 cells/μl of gut macerate). Using a similar artificial infection protocol and counting the parasites in a Neubauer chamber, we observed that, in most of cases, we were unable to detect any parasites in the different segments of the digestive tract and only scored those insects with high parasite loads (S6 Fig). This sampling bias does not produce reliable results to follow insects infected with low parasite densities (<10^4^ parasites/insect), which is frequently observed in natural infections [35]. In addition, in contrast to our results, this method does not allow the detection of parasites in the AM at p.i. times beyond ten days [36] and misses parasites that adhere to insect tissue. This contrasts with our qPCR methodology, which allows the detection of up to 1 parasite/AM, two weeks after the refeeding of infected vectors with low level of parasites (~ 10^4^ parasites) but also cell aggregates. In this respect, it is noteworthy that the presence of parasite nests observed in both AM and PM from day 7 p.i. might function as reservoirs to maintain a basic rate of metacyclogenesis in the vector that is necessary to assure an optimized vectorial competence throughout the life of the infected insect, which can live up to one year under regular feeding conditions [4]. We hypothesized that the presence of small reservoirs of epimastigote parasites in the midgut could be boosted upon blood refeeding, causing the maintenance of the parasite number to a constant level in the different gut compartments with a slight increase in parasite load in the AM and PM at three weeks after infection. The observation that the percentage of metacyclogenesis scored at day 14 p.i. (25.2%) was in a similar range to that scored at day 14 after the second blood feeding (19.1%) supports a control system that balances the relative proportions of dividing epimastigote cells and metacyclic quiescent cells. We postulate that this mechanism that leads to the fine-tuning of the parasite load in each segment of the digestive tract should provide a basic rate of metacyclogenesis that is required to increase the period of vectorial competence.

Previous reports have shown that the kinetics of digestive tract colonization is dependent on several parameters such as the rate of insect infection by metacyclics (or epimastigotes), the efficiency of differentiation from metacyclics to epimastigotes, the multiplication of epimastigotes inside the gut and finally their transformation to metacyclic trypomastigotes. These parameters rely on the species of insect vector, the nature of the gut microbiota [37,38] and the strain or lineage of the infecting parasites [9]. In this respect, our data confirmed the variability in infection level between two different *T*. *cruzi* lineages, although the parasite load curves displayed a similar pattern. Indeed, CL Brener compared to Dm28c exhibited a higher resistance phenotype to lysis, as evidenced by the high percentage of epimastigote forms in the gut (especially in the PM); however, the onset of infection through different segments of the gut followed a similar time course [9]. Our data also showed that independently of the parasite stage used for infecting the insect (epimastigote or trypomastigote forms), the gut colonization followed a similar profile, with a dramatic decrease in parasite number during the first 24 h after infection, which was already noted by Henriques et al. (2012) [13] but at latter times, on the seventh day p.i., and representing 4.5% of ingested trypomastigotes. We show that this decrease is observed in the AM, which represents the first point of contact between the ingested parasites and the vector surface, which is composed of epithelial cells that are devoid of a perimicrovillar membrane [4]. These results suggest that the lysis event may be due to some lytic factor that is able to kill both forms of the protozoan, in particular the trypomastigote, which is the natural form ingested by the insect and is also, despite greater resistance than the epimastigote form to several stress treatments such as complement [39], is also susceptible to lysis in the insect gut. As previously suggested, several factors related to vector immunity could be responsible for this severe reduction in parasite load (*e*.*g*., lectins, antimicrobial peptides (AMPs), and nitric oxide) [40], but other contributing factors could include digestive enzymes and other biochemical factors. In addition, some studies have shown that the microbiota of the triatomine might be involved in the control of the population density of epimastigotes in the gut but this effect is observed at latter times (3 days p.i.) and is not observed for most of the *T*. *cruzi* lineages such as Dm28c [41]. In contrast, our data show that the initial lysis of the Dm28c parasites observed during the first 24 h in the AM is independently of the presence of gut symbionts and may be due to some factors of the innate immune system of the vector that are released during hemoglobin digestion or that are already present in the AM. Several immunity-related components present in the AM might play a central role in the control of the parasite density. Among these factors, AMPs could play a critical role in limiting the infection of the vector, as reported for trypanosomal vectors [42–44]. Other putative trypanolytic factors such as lectins could play a role in determining the outcome of the infection. Lectins were found overexpressed in the AM and PM after a blood meal and have been reported to be involved in the agglutination of parasites [45]. A hypothetical hemolytic factor isolated from AM extracts and supposed to be involved in erythrocyte lysis was reported to lyse the *T*. *cruzi* Y strain more efficiently than the Dm28c strain *in vitro* [41,46]. In this regard, not only the parasite strain but also the source of the blood used to feed insects would be important to take into account to study the lysis event [47]. ROS and nitrogen intermediates could also play a role in the control of *T*. *cruzi* infection in the triatomine gut [48,49]. Regarding the digestive enzymes, although no proteolytic activity was detected in the AM [50, 51], the transcriptome study of the digestive tract of *Rhodnius* revealed an abundance of transcripts encoding aspartyl protease (in particular, cathepsin D-like enzyme) and cysteinyl proteases [52]. In addition, we cannot discard the possibility that some proteolytic enzymes became fully active in the AM through the clipping off of the proprotease precursors by some trypanosomal proteases such as the cruzipain, which is the major cysteine protease in the parasite.

Taken together, our data reveal some unexpected biological features of the colonization of the *R*. *prolixus* midgut by *T*. *cruzi* that may be critical for the investigation of parameters involved in vectorial competence. In laboratories performing xenodiagnosis in chagasic patients or infecting triatomines experimentally, the rate of infection is highly variable and often includes a significant number of insect vectors with no parasite score. Similarly the vector infection rate in endemic regions for Chagas disease is markedly variable, ranging from 5% (Brazil) [53] (Data from *Pan American Health Organization*) to 79% (Bolivia) [54]. It has been proposed that this low infection rate might be due to the lysis of ingested parasites with the blood meal, which, in some cases, could lead to complete parasite lysis with no survival [55]. Our observations confirmed a massive lysis of both epimastigote and trypomastigote parasites during early stages of infection, which could explain the refractivity of the natural vectors to parasite infection or transmission. In addition, we showed that a low rate of infection can be maintained in the vector that would be necessary to increase the vectorial competence for disease transmission.

Finally, the methodology developed in this work for accurately quantifying low levels of parasites in the gut of the triatomine vector constitutes a useful tool to get insight into the dialog between the parasite and its vector. The use of dsRNA for targeting candidate genes involved in the innate immune response of the vector would help in mapping the key factors responsible for parasite killing in the midgut of the vector. At this respect, the use of first instar nymphs that ingest around 20 times less blood than adults would allow us to develop a high-throughput RNAi library screening strategy delivered via bacterial feeding, using BLI as readout, to identify key insect factors that could be involved in the infection output, in particular in the context of the massive lysis event. To conclude, the techniques developed in this study to monitor insect infection constitute a toolbox helpful to screen insect or parasite genes involved in the infection outcome.

## Supporting Information

S1 Fig Representative standard qPCR calibration curves generated from 10-fold serial dilutions of blood spiked with internal loading control (plasmid pLew82) and *T*. *cruzi* Dm28c. The plot shows representative Ct values of the amplification curves generated with primers targeting the *ble* resistance gene or the *T*. *cruzi* repetitive satellite DNA. DNA samples were sequentially diluted 10-fold to obtain from 0.01 to 10 ng of pLew82/50 ng DNA and from 0.02 to 20 ng of *T*. *cruzi* DNA/50 ng DNA considering that approximately 2 x 10^−4^ ng of parasite DNA corresponds to one parasite equivalent. The amplification efficiency of both PCR reactions are indicated.
(TIF)

S2 Fig Standard amplification curves generated from DNA extracted from digestive tract homogenates spiked with epimastigotes of *T*. *cruzi* Dm28c. The plots show representative Ct values of the amplification curves generated with primers targeting *T*. *cruzi* repetitive satellite DNA. DNA samples were sequentially diluted 10-fold to obtain from 0.001 to 10000 parasite equivalents. The results are expressed as parasite equivalents/50 ng DNA. The slope and regression coefficient of the curve and the amplification efficiency are indicated.
(TIF)

S3 Fig *In vivo* time-course evaluation of infection dynamics during the 24 hours after infection of insects with luciferase-expressing parasites. The images show bioluminescence imaging of infected insects at the indicated times.
(TIF)

S4 Fig Evaluation of heparin on *in vitro* growth of *R*. *rhodnii*. (A) *In vitro* bacteria growth was monitored in the presence (+) or absence (-) of heparin (2.5 units/ml) during 72 hours by OD measurement at 600 nm. OD values (mean values, n = 6) were plotted versus time (hours). (B) Bacteria cultivated in the presence or absence of heparin during 72 hours were serially diluted and plated on LB agar plates for colony forming units (CFU) counting. Representative plates are shown.
(TIF)

S5 Fig Comparison of time-course development of epimastigotes and trypomastigotes expressing luciferase in the digestive tract of nymphs during the first 24 h p.i., in the presence or absence of *R*. *rhodnii*. The nymhs were fed on rabbit blood containing 1 x 10^7^ epimastigotes (Epi) or trypomastigotes (Trypo) of *T*. *cruzi* (Dm28c) per ml containing or not 5 x 10^5^ *Rhodococcus rhodnii* per ml of blood. The time-course monitoring of infection was assessed at 6 h (0 d) and 24 h after the infection (1 d) by bioluminescence imaging of parasites constitutively expressing luciferase. The pictures were taken from three representative nymphs among the eight infected in each condition.
(TIF)

S6 Fig Evaluation of infection dynamics by counting the parasites in a Neubauer chamber. The insects were fed on blood containing 10^7^ epimastigotes/ml and dissected after feeding at the indicated times. The anterior midgut (AM), posterior midgut (PM) and hindgut (H) were individually homogenized in 50 μl of PBS. The parasites in a 10-μl homogenate aliquot were counted.
(TIF)

S1 Video *In vivo* imaging of GFP-tagged parasites in AM of *R*. *prolixus* at 7 days p.i. (MP4)

S2 Video *In vivo* imaging of GFP-tagged parasites in PM of *R*. *prolixus* at 7 days p.i. (MP4)

S3 Video *In vivo* imaging of GFP-tagged parasites in H of *R*. *prolixus* at 7 days p.i. (MP4)
