# Supplementary figures and images for: Monitoring of the Parasite Load in the Digestive Tract of Rhodnius prolixus by Combined qPCR Analysis and Imaging Techniques Provides New Insights into the Trypanosome Life Cycle
Source: PLoS Negl Trop Dis. 2015 Oct 23;9(10):e0004186. doi: 10.1371/journal.pntd.0004186 (PMC4619730; doi:10.1371/journal.pntd.0004186)

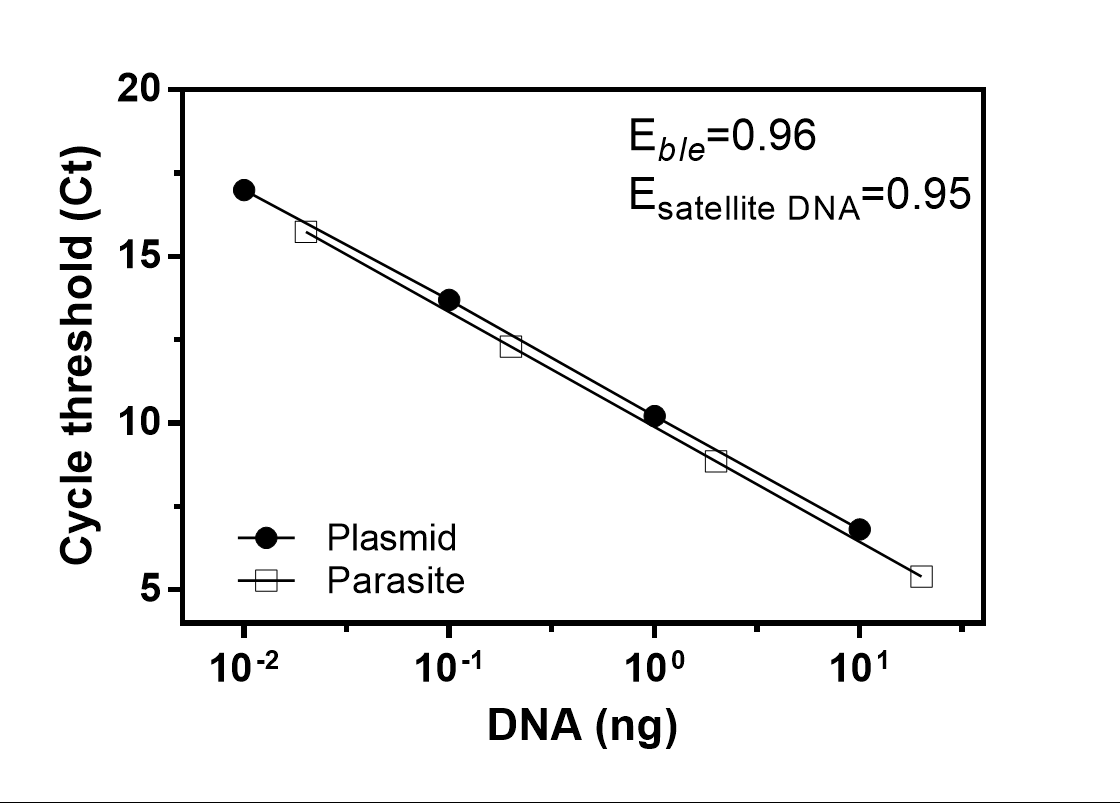

Supplement: S1 Fig — The plot shows representative Ct values of the amplification curves generated with primers targeting the ble resistance gene or the T. cruzi repetitive satellite DNA. DNA samples were sequentially diluted 10-fold to obtain from 0.01 to 10 ng of pLew82/50 ng DNA and from 0.02 to 20 ng of T. cruzi DNA/50 ng DNA considering that approximately 2 x 10^−4^ ng of parasite DNA corresponds to one parasite equivalent. The amplification efficiency of both PCR reactions are indicated. (TIF) [file pntd.0004186.s001.tif]

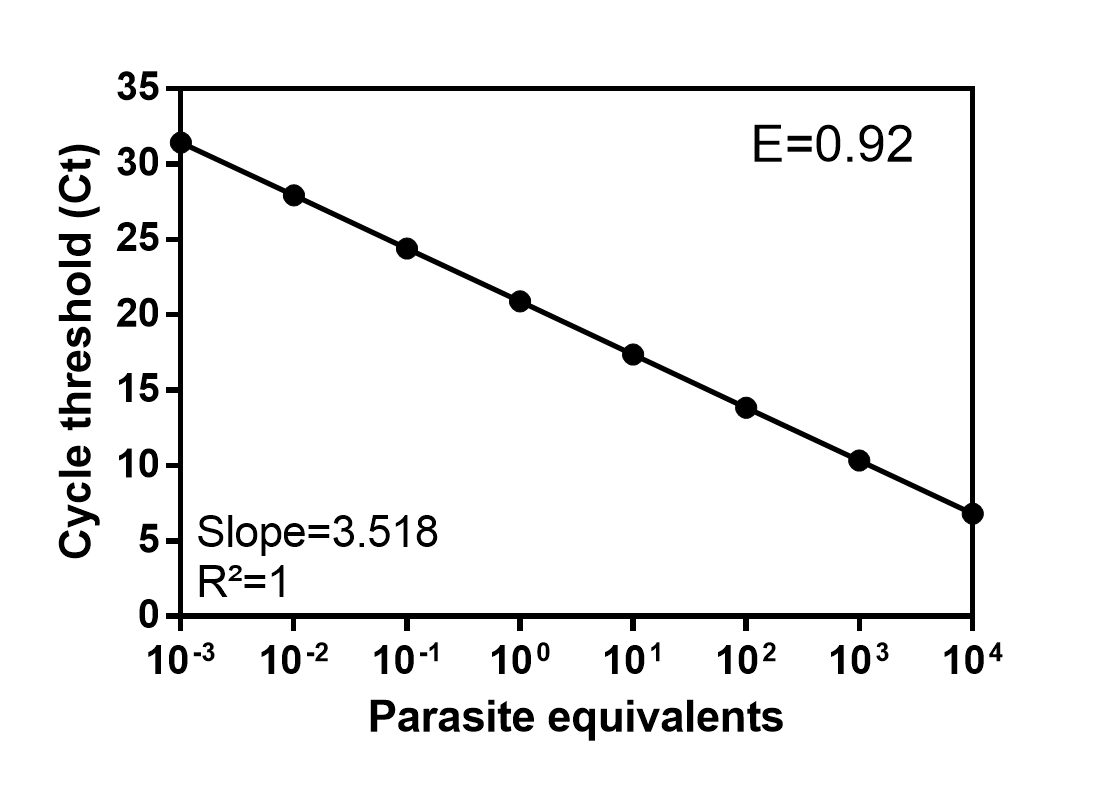

Supplement: S2 Fig — The plots show representative Ct values of the amplification curves generated with primers targeting T. cruzi repetitive satellite DNA. DNA samples were sequentially diluted 10-fold to obtain from 0.001 to 10000 parasite equivalents. The results are expressed as parasite equivalents/50 ng DNA. The slope and regression coefficient of the curve and the amplification efficiency are indicated. (TIF) [file pntd.0004186.s002.tif]

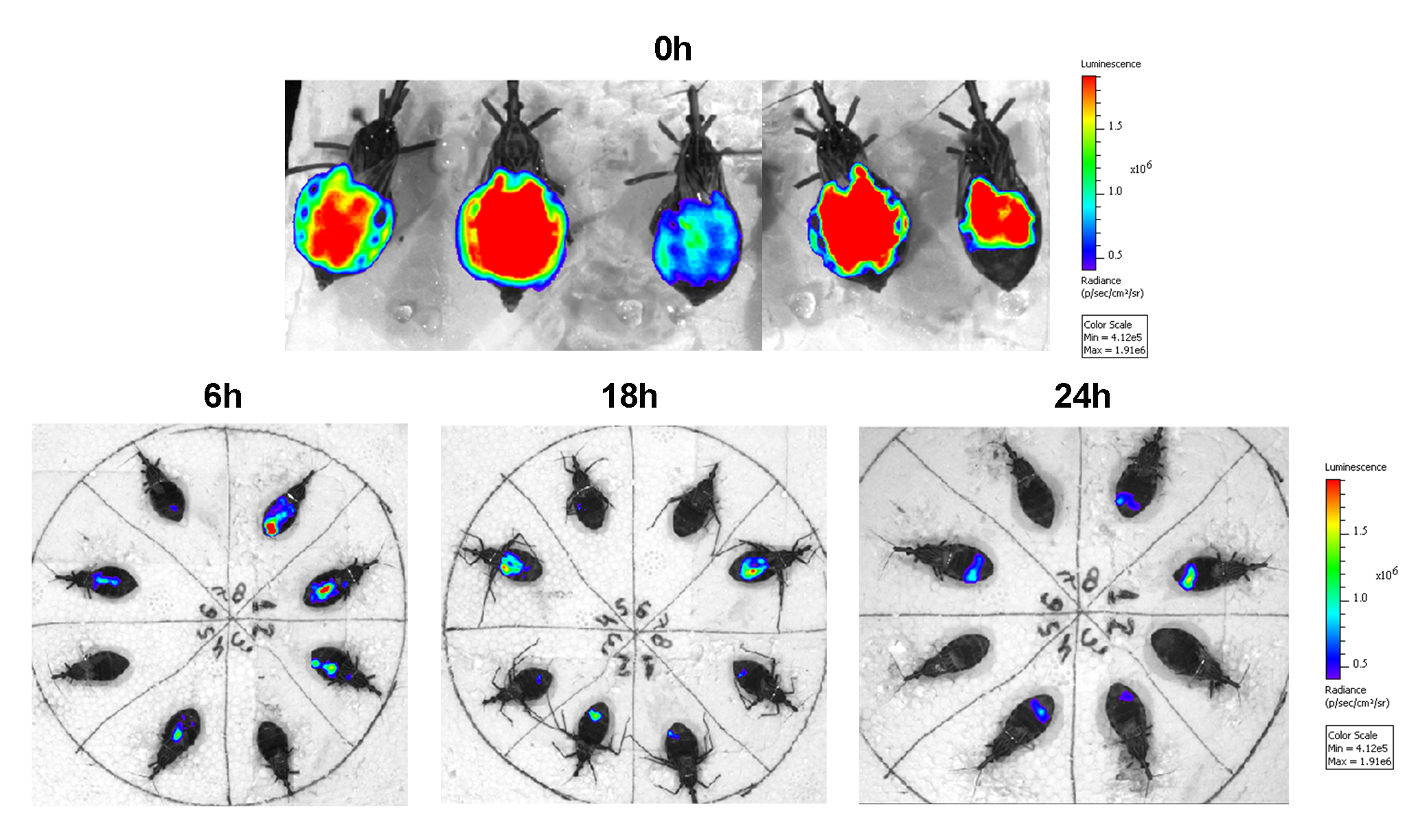

Supplement: S3 Fig — The images show bioluminescence imaging of infected insects at the indicated times. (TIF) [file pntd.0004186.s003.tif]

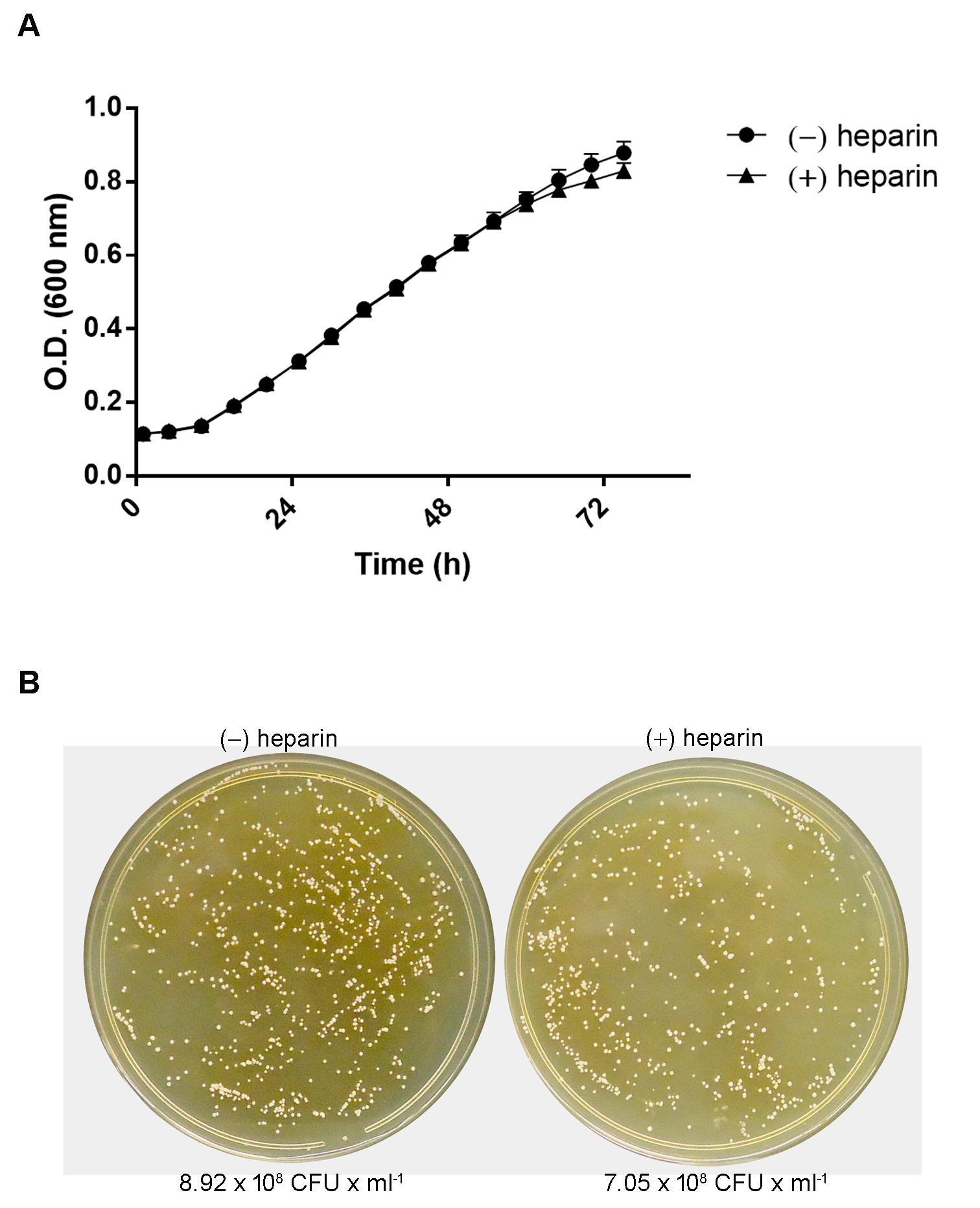

Supplement: S4 Fig — (A) In vitro bacteria growth was monitored in the presence (+) or absence (-) of heparin (2.5 units/ml) during 72 hours by OD measurement at 600 nm. OD values (mean values, n = 6) were plotted versus time (hours). (B) Bacteria cultivated in the presence or absence of heparin during 72 hours were serially diluted and plated on LB agar plates for colony forming units (CFU) counting. Representative plates are shown. (TIF) [file pntd.0004186.s004.tif]

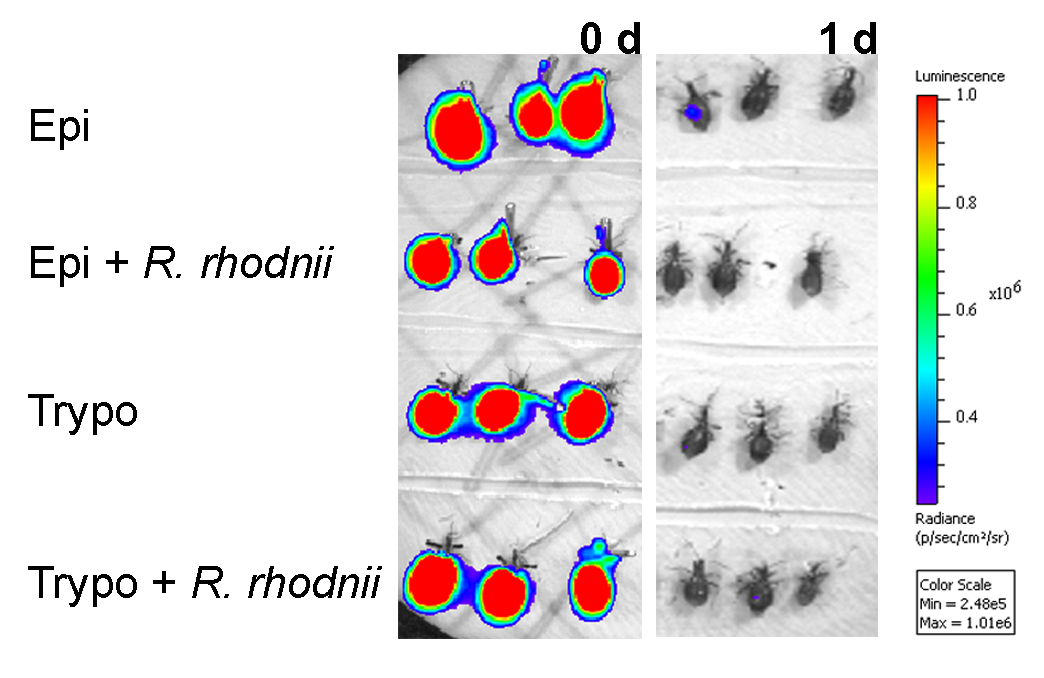

Supplement: S5 Fig — The nymhs were fed on rabbit blood containing 1 x 10^7^ epimastigotes (Epi) or trypomastigotes (Trypo) of T. cruzi (Dm28c) per ml containing or not 5 x 10^5^ Rhodococcus rhodnii per ml of blood. The time-course monitoring of infection was assessed at 6 h (0 d) and 24 h after the infection (1 d) by bioluminescence imaging of parasites constitutively expressing luciferase. The pictures were taken from three representative nymphs among the eight infected in each condition. (TIF) [file pntd.0004186.s005.tif]

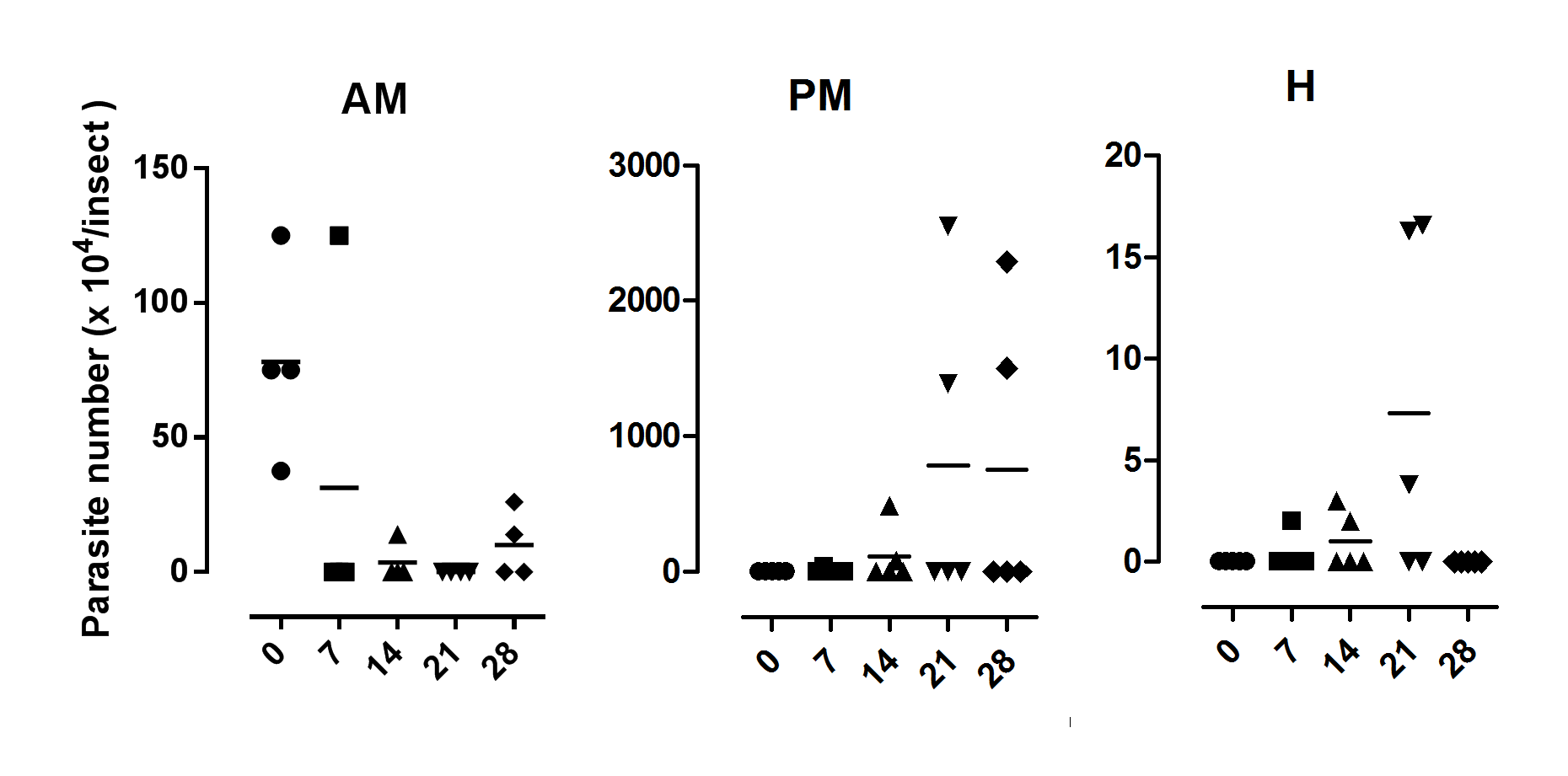

Supplement: S6 Fig — The insects were fed on blood containing 107 epimastigotes/ml and dissected after feeding at the indicated times. The anterior midgut (AM), posterior midgut (PM) and hindgut (H) were individually homogenized in 50 μl of PBS. The parasites in a 10-μl homogenate aliquot were counted. (TIF) [file pntd.0004186.s006.tif]
